# Rice and shine: a review of miRNA-mediated responses to abiotic stress in *Oryza sativa*, from drought to ionizing radiation

**DOI:** 10.3389/fpls.2026.1828169

**Published:** 2026-06-10

**Authors:** Serena Bordignon, Nele Horemans, Tina Kyndt, Gustavo Turqueto Duarte

**Affiliations:** 1 Department of Biotechnology, Faculty of Bioscience Engineering, Ghent University, Ghent, Belgium; 2 Biosphere Impact Studies, Belgian Nuclear Research Centre (SCK CEN), Mol, Belgium; 3 Centre for Environmental Research, University of Hasselt, Hasselt, Belgium

**Keywords:** abiotic stress, ionizing radiation, miRNA, *Oryza sativa*, stress responses

## Abstract

MicroRNAs (miRNAs) are a class of 20–24 nucleotides-long small non-coding RNAs that regulate gene expression at post-transcriptional level via directing cleavage or translational repression of complementary mRNA targets. In plants, in addition to regulating biological processes essential for proper growth and development, miRNAs are also involved in fast responses to stress. Rice (*Oryza sativa*) is one of the most valuable crop species, feeding over half of the global population; however, its productivity is severely affected by unfavorable environmental and climatic conditions, including drought, salinity, temperature extremes, heavy metal toxicity, and nutrient deficiencies. In this context, numerous stress-responsive miRNAs have been identified in rice, highlighting their contribution to cellular homeostasis, developmental adjustment, and stress acclimation. This review provides a comprehensive overview of miRNA biogenesis in plants and their roles in rice responses to major abiotic stresses. Special emphasis is given to miRNA-mediated regulation under genotoxic stress, particularly that induced by ionizing radiation. Overall, available evidence suggests that rice miRNA responses combine recurrent regulatory modules shared across stresses with context-dependent, stress-specific patterns, while miRNA involvement in genotoxic stress responses remains comparatively underexplored.

## 1 Introduction

Rice (*Oryza sativa*) is the staple food for more than half of the world’s population, especially in East and South Asia, the Middle East, the West Indies and Latin America (Sharif et al., 2014). Together with wheat and maize, it accounts for 50% of the global caloric intake, with rice alone accounting for 23%, wheat 17%, and maize 9% (Kole, 2006). Besides its economic importance, rice also represents a model organism for molecular research because of the relatively small size of its genome when compared to other cereals, the large germplasm collection, the wide array of molecular genetic resources, and the availability of efficient transformation protocols (Paterson et al., 2005; Zafar et al., 2020; Rengasamy et al., 2024; Dash et al., 2025). However, even though rice cultivation has been established since ancient times, unfavorable environmental and climatic conditions continue to impact growth and productivity of this valuable food source (Saud et al., 2022; Sarma et al., 2023). Among these, abiotic stresses such as drought (Ma et al., 2024), high salinity (Saleem et al., 2025), cold (Shahzad et al., 2024), heat (Xing et al., 2024), heavy metal contamination (Arif et al., 2019), and nutrient deficiency (Shrestha et al., 2020) are the most significant. In response to these constant challenges, plants have evolved complex regulatory mechanisms to cope with stresses that affect their development, genome stability, and yield (Abhishek et al., 2022; Taria et al., 2022). Among the different mechanisms central to stress response in plants, post-transcriptional regulation of gene expression has been suggested as one of the most important for maintaining cellular homeostasis under stress conditions (Sunkar et al., 2012). In this context, microRNAs (miRNAs), a class of endogenous small non-coding RNAs generated from longer precursor molecules modulate gene expression post-transcriptionally through sequence complementarity by directing target mRNA cleavage, translational repression or DNA methylation (Song et al., 2019). In plants, miRNA-mediated gene regulations include the maintenance of genome integrity, control of development and metabolism (Bartel and Bartel, 2003; Vaucheret et al., 2004; Willmann and Poethig, 2007; Djami-Tchatchou et al., 2017; Li et al., 2017c; Gao et al., 2021), as well as rapid responses to environmental stresses (Khraiwesh et al., 2012; Shriram et al., 2016; Djami-Tchatchou et al., 2017; Li et al., 2017c; Brant and Budak, 2018; Gao et al., 2021). In rice, pioneering work in the study of miRNAs was conducted by Wang et al. (Wang, 2004) and Sunkar et al (Sunkar et al., 2005). Since then, advances in sequencing technologies have greatly expanded the detection and recognition of miRNAs in rice, with hundreds currently cataloged in the public universal miRNA database miRBase (release 22.1, https://mirbase.org/) (Kozomara et al., 2019).

**Figure 1:**
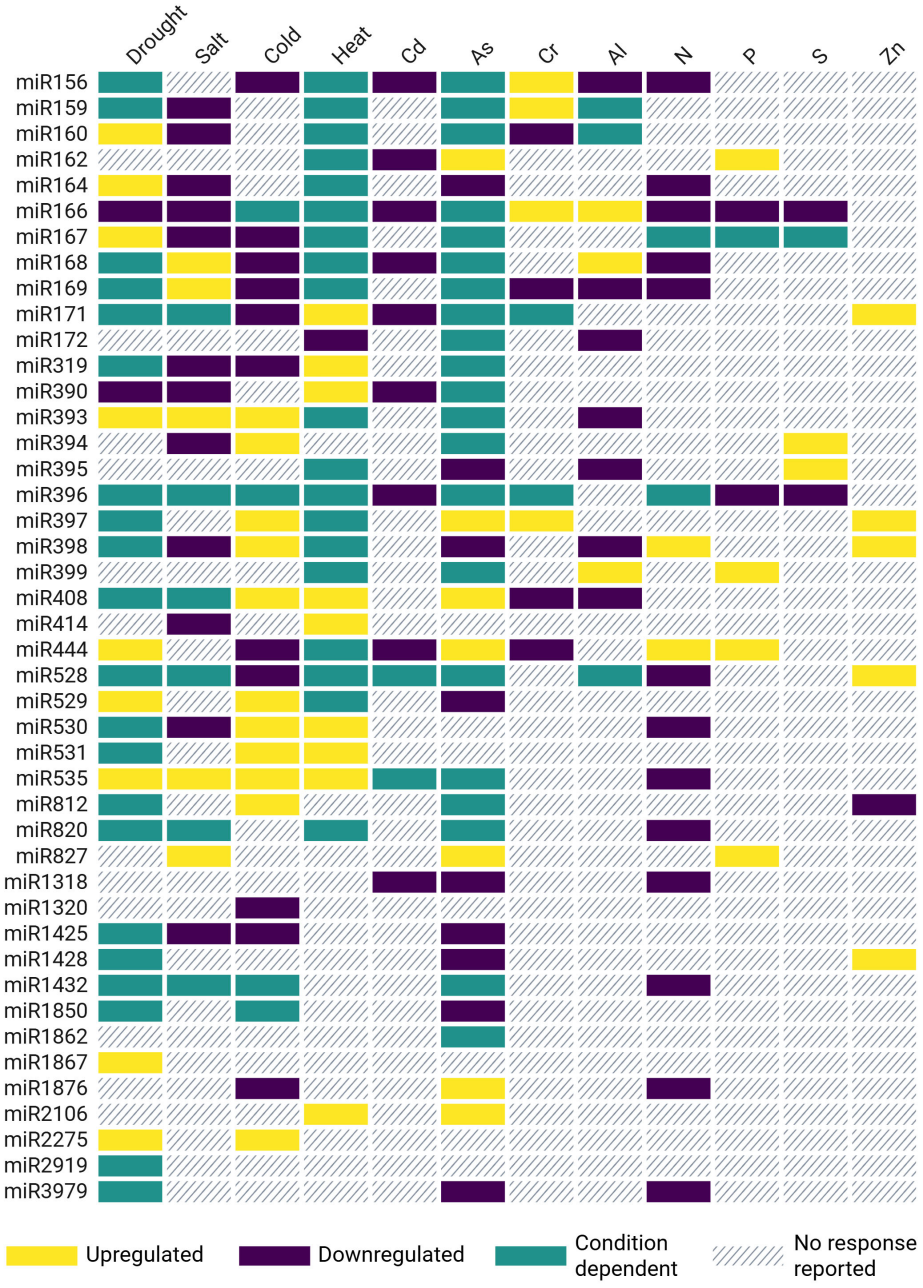
Differential expression of rice miRNAs with validated target genes under various abiotic stress conditions. Rows represent individual miRNA families, and columns represent abiotic stress conditions (i.e., drought, salt, cold, heat, heavy metals, nutrient deficiencies). Individual heavy metals and nutrient deficiencies are shown as separate columns. Only miRNA families with experimentally validated target genes are included. Colors indicate miRNA expression responses: yellow, upregulated; purple, downregulated; green, condition-dependent regulation; gray, no reported response. **Supplementary Table 1** provides a complete list of stress-responsive miRNAs, including those without validated target genes, using the same matrix layout and additionally reporting validated target genes and corresponding literature references. BioRender. Bordignon, S. (2026) https://BioRender.com/866nxxa.

In this review, we provide a comprehensive overview of miRNAs biogenesis in plants and their involvement in the most significant abiotic stress responses in rice, including drought, salinity, temperature extremes, heavy metals and nutrient deficiencies (**Figure 1**; **Supplementary Table 1**). As a new perspective, focus is given to miRNAs-dependent responses to genotoxic stresses in plants, especially those induced by ionizing radiation (IR). In this context, we also examine the involvement of rice miRNAs in DNA repair pathways and oxidative stress responses, which may contribute to the overall adaptation to IR stress.

## 2 Mechanisms of miRNA biogenesis in plants

MicroRNAs are a type of small (20–24 nucleotides) non-coding RNA molecules that are encoded by endogenous genes and regulate gene expression post-transcriptionally through sequence complementarity (Carthew and Sontheimer, 2009; Voinnet, 2009; Zhang and Lu, 2011; Yu et al., 2017; Li et al., 2024). Since the discovery of the first miRNA, lin-4, and its target in *Caenorhabditis elegans* (Lee et al., 1993), they have also been found in most *Eukaryotes*, including plants. The first plant miRNA was identified in *Arabidopsis thaliana* in 2002 (Reinhart et al., 2002). As of the latest release of the miRBase database (v22.1), hundreds of miRNAs have been reported in different plant species, including *A. thaliana* (428), *O. sativa* (757), *Medicago truncatula* (375), *Zea mays* (321), *Sorghum bicolor* (242), and *Vitis vinifera* (186) (https://mirbase.org/) (Kozomara et al., 2019).

**Figure 2:**
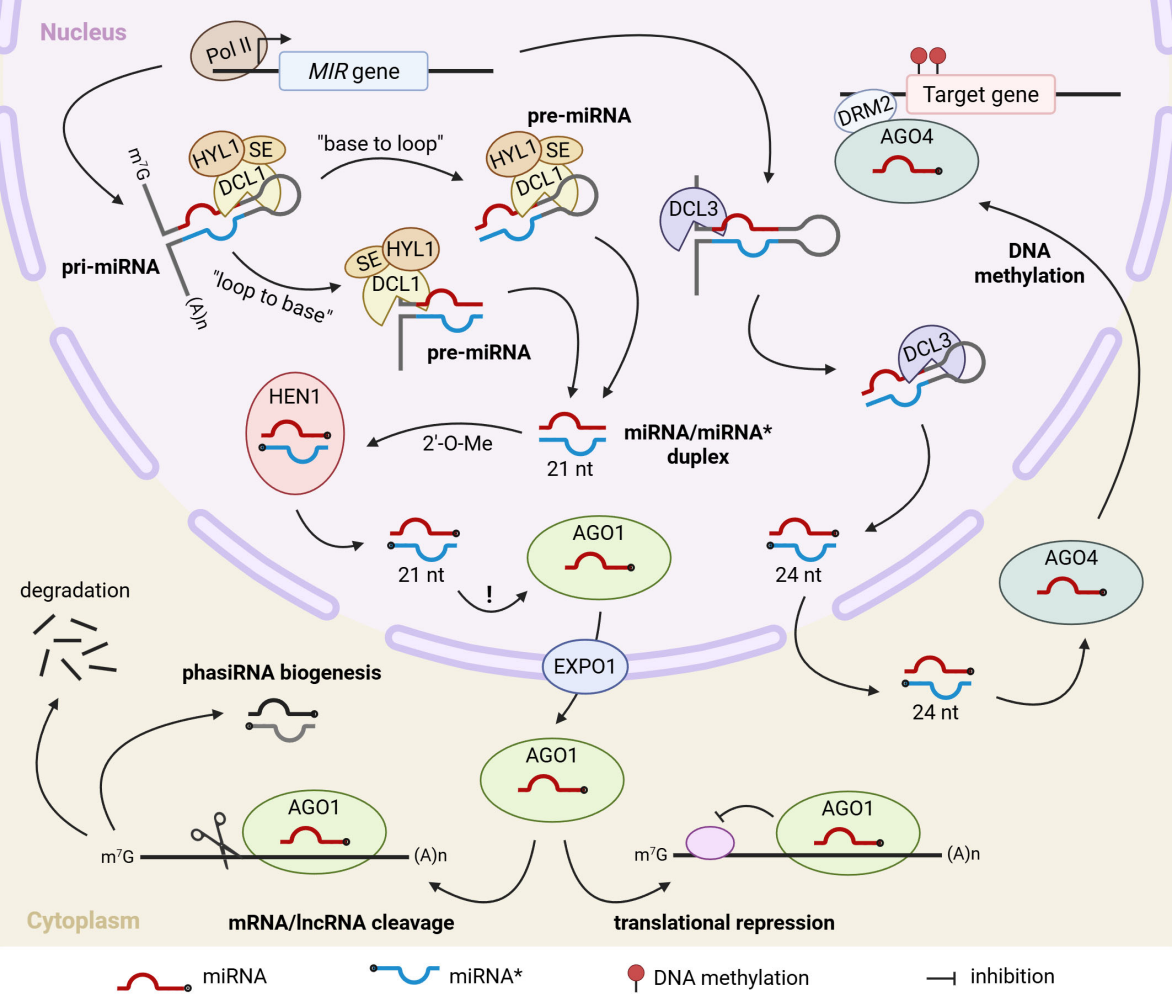
Schematic representation of miRNA biogenesis, RISC assembly, and miRNA-mediated gene regulation in plants. miRNA genes (*MIRs*) are first transcribed by RNA Polymerase II (Pol II) into primary miRNAs (pri-miRNAs) in the nucleus. pri-miRNAs are then recognized by the dicing complex, composed of DCL1, HYL1 and SE, and processed by two sequential DCL1 cleavages: first to a 60–300 nt stem-loop precursor (pre-miRNA; processed base-to-loop or loop-to-base depending on precursor length), then to a 21–24 nt miRNA/miRNA* duplex. HEN1 then methylates the 3’ ends of miRNA duplexes via 2’-O-methylation. Next, the 21 nt mature miRNA strands are loaded into AGO1 to form the RNA-induced silencing complex (RISC), together with the molecular chaperones HSP90 and CYP40. RISC complex is then exported to the cytoplasm by EXPO1, where it mediates target mRNA cleavage or translational repression. miRNA-guided cleavage of target transcripts, including mRNAs and lncRNAs, may result in transcript degradation via the exosome complex and the exoribonuclease XRN4 or initiate the biogenesis of phasiRNAs. Alternatively, 24 nt miRNAs are loaded into AGO4 and guide DNA methylation by DRM2. 2’-O-Me, 2’-O-methylation; AGO1/4, ARGONAUTE 1/4; DCL1/3, DICER-LIKE 1/3; DRM2, DOMAINS REARRANGED METHYLTRANSFERASE 2; HEN1, HUA ENHANCER 1; HYL1, HYPONASTIC LEAVES 1; miRNA, microRNA; miRNA*, miRNA passenger strand; MIR, miRNA gene; RISC, RNA-induced silencing complex; nt, nucleotide; phasiRNA, phased small interfering RNAs; Pol II, RNA Polymerase II; pri-miRNA, primary microRNA; SE, SERRATE. Symbols: black dot, miRNA methylation; exclamation point, non-canonical loading of miRNA* into AGO1. Created in BioRender. Bordignon, S. (2026) https://BioRender.com/u50lfu1.

A schematic representation of miRNA biogenesis is shown in **Figure 2**. While some plant miRNAs originate from introns of protein-coding genes or other non-coding RNAs including tRNAs, rRNAs and snRNA (Abdelfattah et al., 2014; Alves et al., 2017), the majority of plant miRNAs are encoded by independent transcription units (*MIR* genes) located in intergenic regions (Parizotto et al., 2004; Millar and Waterhouse, 2005; Rajagopalan et al., 2006; Budak and Akpinar, 2015; Knop et al., 2016). It is estimated that between 1 and 2% of a plant’s genes are *MIR* genes, with many of them existing as families (Li and Mao, 2007; Zhang et al., 2008; Nozawa et al., 2012; Budak and Akpinar, 2015). These genes are transcribed by DNA-dependent RNA polymerase II (Pol II) into primary miRNA transcripts (pri-miRNAs), which are usually between hundreds and thousands of nucleotides long and undergo a series of transcriptional modifications including 5′ capping and 3′ polyadenylation (Kurihara and Watanabe, 2004; Lee et al., 2004; Bologna et al., 2013). Since most pri-miRNAs contain a premature stop codon and are potential targets of nuclear RNA machinery, their protection from degradation during co-transcriptional processing is likely critical for proper miRNA balance (Li and Yu, 2021). In addition, alternative splicing of pri-miRNAs has also been reported to affect miRNA balance by reducing the cleavage efficiency of the miRNA processing machinery. In rice, for example, *MIR528* contains two alternative splicing sites that lead to the generation of two forms of pri-miR528 with different processing efficiencies (Yang et al., 2019). Throughout development, their individual expression levels change, leading to differential accumulation of miR528 (Yang et al., 2019).

The pri-miRNAs, folded into a partially complementary paired hairpin structure, are then recognized by the dicing complex (**Figure 2**), which is visualized as a D-body in the nucleus (Fang and Spector, 2007). Assisted by the double-stranded RNA-binding protein HYPONASTIC LEAVES1 (HYL1) (Han et al., 2004; Vazquez et al., 2004; Kurihara et al., 2006), the C2H2-zinc finger structural protein SERRATE (SE) (Lobbes et al., 2006; Dong et al., 2008; Machida et al., 2011), and the cap-binding protein complex (CBC), DICER-like RNase III endonuclease 1 (DCL1) cleaves the pri-miRNAs to form the precursor miRNAs (pre-miRNAs), which are 60 to 300 nt long and have a stem-loop structure (Kim, 2005; Voinnet, 2009; Wang et al., 2019). The resulting pre-miRNAs are further processed by the dicing complex to form miRNA duplexes consisting of a guide strand (mature miRNA) and a passenger strand (miRNA*) (Voinnet, 2009; Song et al., 2019). Processing can involve single or multiple DCL1-mediated cleavage events, depending on the precursor length. Short miRNA precursors are processed by DCL1 through a single cleavage, either from the loop to the base or from the base to the loop. In contrast, long miRNA precursors are processed by successive DCL1-mediated cleavages, either from the loop to the base, from the base to the loop, or bidirectionally (Addo-Quaye et al., 2009; Bologna et al., 2009; Bologna et al., 2013; Zhu et al., 2013).

The nascent miRNA/miRNA* duplexes generated by DCL-mediated processing exhibit 2 nt 3′ overhangs on both strands. In addition, each strand possesses a 5′ end phosphate and a 3′ end hydroxyl groups, which are 2′-O-methylated by the methyltransferase HUA ENHANCER 1 (HEN1) to prevent their uridylation and subsequent degradation (**Figure 2**) (Li et al., 2005; Yu et al., 2005; Yang, 2006). The guide strands of miRNA/miRNA* duplexes, which are selected due to their lower thermodynamic stability at the 5′ end, are then incorporated into ARGONAUTE (AGO) proteins, which, together with the molecular chaperones HEAT SHOCK PROTEIN 90 (HSP90) (Iki et al., 2010) and CYCLOPHILIN 40 (CYP40) (Iki et al., 2012), form the RNA induced silencing complex (RISC) (**Figure 2**) (Baumberger and Baulcombe, 2005; Eamens et al., 2009). The passenger strands (miRNA*) are usually degraded. However, under specific circumstances, for example during plant development and/or under stress conditions, they can replace the guide strands and be loaded into RISC, becoming the dominant strand (Wen-wen et al., 2014; Xu et al., 2018). Several studies have indicated that the recruitment and binding of miRNAs to specific AGO proteins is directed by the nature of the 5′ nucleotides: miRNAs with a 5′-terminal uridine are mainly incorporated into AGO1, miRNAs with a 5′-terminal adenosine are largely associated with AGO2, and miRNAs with a 5′-terminal cytosine are associated with AGO5 (Mi et al., 2008; Takeda et al., 2008). In addition, the loading of miRNAs into AGO proteins is also affected by the bulges in the duplex structures: miRNA duplexes with central mismatches are incorporated into AGO1, whereas miRNA duplexes without central mismatches are associated with AGO2 (Ren et al., 2014). Recent studies have shown that RISC assembly occurs in the nucleus and is then exported to the cytosol by EXPORTIN 1 (EXPO1), a process facilitated by the Nucleoporin 1 (NUP1)/Transcription-coupled Export 2 complex (TREX-2) (Bologna et al., 2018; Zhang et al., 2020a). In contrast, HASTY (HST), the *A. thaliana* ortholog of human EXPORTIN 5, which was considered the candidate protein for the export of miRNAs from the nucleus to the cytoplasm for the past two decades, has recently been reported to participate in the transcription and processing of pri-miRNAs by interacting with DCL1 and MED37 (Cambiagno et al., 2021). Therefore, HST functions in the miRNA pathway independently of its cargo-exporting activity, and *hst* mutants exhibit normal subcellular distribution of miRNAs (Cambiagno et al., 2021). In rice, the Xpo1 domain protein CROWN ROOT DEFECT 1 (CRD1), the ortholog of *A. thaliana* HST, was found to be essential for maintaining normal miRNA levels, as the *crd1* mutant contained significantly reduced miRNA levels in both the nucleus and the cytoplasm (Zhu et al., 2019). This finding also contrasts with the function of CRD1 as a nuclear exporter, as miRNAs would be expected to accumulate in the nucleus of *crd1* (Zhu et al., 2019). However, the detailed mechanism behind CRD1 function in miRNA stability still needs further investigation.

The miRNA biogenesis pathway itself is subject to feedback regulation. DCL1 abundance is fine-tuned by two negative feedback mechanisms. First, DCL1 mRNA is cleaved by miR162, a miRNA generated by DCL1 itself (Xie et al., 2003). Second, DCL1-mediated processing of *MIR838*, an intronic *MIR* gene derived from the 14^th^ intron of DCL1 pre-mRNA, results in unproductive fragments of DCL1 pre-mRNA (Rajagopalan et al., 2006). The expression of *SE* and *AGO1* is also subject to feedback regulation by miR168 and miR863, respectively (Vaucheret et al., 2004; Niu et al., 2016).

## 3 Mechanisms of miRNA-mediated regulation in plants

Once incorporated in RISC, and unlike most animal miRNAs, plant miRNAs predominantly direct endonucleolytic cleavage of their target mRNAs, but can also inhibit their translation (Pegler et al., 2019; Choudhary et al., 2021; Gao et al., 2021). Besides these two major mechanisms, plant miRNAs can direct DNA methylation and are involved in the biogenesis of phase secondary small interfering RNAs (phasiRNAs) and in miRNA-long non-coding RNA (lncRNA) interactions (**Figure 2**) (Wu et al., 2010; Liu et al., 2020; Meng et al., 2021).

### 3.1 Target cleavage

The miRNA transcript cleavage process is mediated by the endonuclease activity of the AGO1 protein and occurs at the phosphodiester bond opposite the 10^th^ and 11^th^ nucleotides of the miRNA through near-perfect sequence complementarity (Llave et al., 2002; Baumberger and Baulcombe, 2005; Qi et al., 2005). Subsequently, the exosome complex and the exoribonuclease XRN4 rapidly degrade the cleaved target mRNAs, leading to a robust downregulation of target gene expression (Souret et al., 2004; Sieburth and Vincent, 2018).

### 3.2 Translation inhibition

The miRNA translation repression process impairs the ability of the ribosome to initiate translation or progress along the mRNA strand, effectively blocking the synthesis of functional proteins (Li et al., 2024). In contrast to transcriptional cleavage, the target mRNA remains intact, meaning that translation can resume once the repression is removed (Alvarez et al., 2006; Brosnan and Mitter, 2021). Several studies have reported that HYL1, also known as DRB1, is involved in miRNA-mediated cleavage of target mRNA, while DRB2, another member of the DRB family that interacts with DCL1, is involved in miRNA-mediated translational repression (Reis et al., 2015).

### 3.3 DNA methylation

In addition to post-transcriptional gene silencing, plant miRNAs are capable of transcriptional gene silencing. Although DCL1-dependent 21-nt long miRNAs are mainly produced in plants, in rice a proportion of the pre-miRNAs can be processed by DCL3 into 24-nt long miRNAs (long miRNAs, lmiRNAs), which are loaded into AGO4 effector, and direct DNA methylation (Wu et al., 2010; Campo et al., 2021).

### 3.4 phasiRNA biogenesis

While miRNAs are generated from RNA hairpin precursors, their activity can also trigger the production of another class of small ncRNAs: phased small interfering RNAs (phasiRNAs). In plants, miRNA-guided cleavage of target transcripts, including mRNAs and lncRNAs, can initiate phasiRNAs biogenesis. Like miRNAs, phasiRNAs are typically 21 or 24 nt in length and can mediate target mRNA degradation or epigenetic regulation (e.g., DNA methylation) (Liu et al., 2020). Twenty-one-nt phasiRNAs are generated by RDR6/DCL4 and act mainly post-transcriptionally, whereas 24-nt reproductive phasiRNAs (in grasses) are DCL5-dependent and are linked to RdDM-like epigenetic control (Liu et al., 2020).

### 3.5 miRNA-lncRNA interaction

Recently, miRNA-lncRNA interactions have emerged as an additional regulatory layer of gene expression in plants, crucial for development, physiology and responses to biotic and abiotic stresses (Meng et al., 2021; Li et al., 2023). While miRNAs can target lncRNAs and trigger phasiRNA biogenesis, lncRNAs can also act as miRNA precursors or regulate miRNA accumulation and activity at both transcriptional and post-transcriptional levels (Pouch-Pélissier et al., 2008; Li et al., 2016b; Li et al., 2020; Jiang et al., 2020; Meng et al., 2021). Among the different mechanisms underlying miRNA-lncRNA interactions, target mimicry is one of the most relevant. Through sequence complementarity, lncRNAs containing endogenous target mimic sites can sequester miRNAs, thereby reducing their activity on target mRNAs (Meng et al., 2021). For instance, in rice, *MIKKI*, a root-specific retrotransposon-derived lncRNA, binds to miR171 and acts as a decoy to inhibit miRNA-mediated cleavage of *SCARECROW-Like* (*SCL*) mRNAs, thereby leading to increased cell elongation in the root (Cho and Paszkowski, 2017). Furthermore, the interaction between miRNAs and lncRNAs seem to be critical for controlling plant parasitic nematode infections (Verstraeten et al., 2021). Interestingly, some core components of the miRNA biogenesis machinery, including SERRATE, CBP20 and CBP80, also participate in the splicing of intron-containing lncRNAs, further highlighting the close functional interplay between these regulatory RNA pathways (Liu et al., 2012).

## 4 miRNA-mediated responses to major abiotic stresses in rice

In plants, miRNAs are involved in the regulation of a wide range of biological processes including the maintenance of genome integrity, development and metabolism (Bartel and Bartel, 2003; Vaucheret et al., 2004; Willmann and Poethig, 2007; Djami-Tchatchou et al., 2017; Li et al., 2017c; Gao et al., 2021). Moreover, miRNAs are essential for adaptive responses to environmental cues, among which several abiotic-related stress signals, such as drought, high salinity, extreme temperature, heavy metal contamination, and nutrient deficiency.

Considering the great importance of rice as a staple food crop and the significant impact of abiotic stressors on its growth and productivity, understanding how rice miRNAs respond to different abiotic stressors and how they affect gene expression and other regulatory mechanisms related to stress response is crucial for the development of stress-resistant rice varieties. As of the latest release, 738 and 699 mature miRNAs for rice are present in the miRNA database miRBase (https://mirbase.org/) (Kozomara et al., 2019) and in the Plant miRNA Encyclopedia (PmiREN) (Guo et al., 2020), respectively, among which several are stress-related. Further details on the roles of *validated* miRNAs in specific abiotic stress responses in rice are presented in the following sections and can be found in **Figure 1** and **Supplementary Table 1**.

### 4.1 Drought stress

Drought is one of the most severe environmental constraints on agriculture worldwide, particularly for rice, as paddy field crops are particularly susceptible to water scarcity (Dixit et al., 2014). Several studies revealed that drought regulates the expression of miRNAs in both monocot and dicot plants including rice (Zhou et al., 2010), tobacco (Frazier et al., 2011; Guo et al., 2017), barley (Hackenberg et al., 2015; Ferdous et al., 2017), common bean (Wu et al., 2017a), soybean (Kulcheski et al., 2011), maize (Wei et al., 2009; Aravind et al., 2017), *A. thaliana* (Liu et al., 2008), poplar (Qin et al., 2011), tomato (Liu et al., 2017b; Liu et al., 2017c), wheat (Kantar et al., 2011; Liu et al., 2017a), and sorghum (Katiyar et al., 2015).

In a genome-wide study, Zhou et al. identified multiple drought-responsive miRNAs in rice across different developmental stages, among which four osa-miRNA families (miR168, miR171, miR172 and miR397) were downregulated and two (miR169 and miR319) were upregulated (**Figure 1**; **Supplementary Table 1**) (Zhou et al., 2010). High-throughput sequencing of small RNAs in rice roots exposed to either full drought or a split-root system, in which one half was fully watered and the other half was water-deprived, enabled the identification of 52 drought-responsive miRNAs and 61 drought-signaling-responsive miRNAs (Bakhshi et al., 2016) (**Figure 1**; **Supplementary Table 1**). Notably, downregulation of miR159 under drought stress and drought signaling conditions increased the expression of the GAMYB transcription factor, a positive regulator of abscisic acid (ABA) signaling and a negative regulator of growth, thereby contributing to growth inhibition. Conversely, upregulation of miR159 under wet signaling conditions triggered an opposite effect (Bakhshi et al., 2016). Interestingly, the opposite behavior of miR159 under split-root conditions suggests that miRNA expression may shift depending on the spatial distribution of water availability, allowing the plant to discriminate between local water shortage and systemic stress perception. Additionally, downregulation of miR528 under drought and drought signaling conditions suggested a role in superoxide dismutase (SOD) regulation and reactive oxygen species (ROS) detoxification (Bakhshi et al., 2016), consistent with earlier findings showing increased SOD expression in drought-stressed rice under similar experimental conditions (Mirzaei et al., 2014).

The functions of specific miRNAs in rice drought stress and tolerance have been further uncovered through functional studies, with a total of 44 miRNA families reported as differentially expressed (**Figure 1**; **Supplementary Table 1**). Fang et al. showed that the conserved miR164-targeted *NAC* genes (*OsOMTN2*, *OsOMTN3*, *OsOMTN4*, and *OsOMTN6*), which participate in the regulation of organ boundaries and normal plant development, may act as negative drought tolerance regulators, as their overexpression increased the plant sensitivity to drought stress at the reproductive stage (Fang et al., 2014). In contrast, overexpression of a miR164-resistant *OsOMTN2* mutant gene improved rice plant architecture and yield, as well as drought and salt tolerance, which may be partly due to the direct interaction of *OsOMNT2* with the promoter of *OsLEA3* (Jiang et al., 2019). Because *OsMNT2* regulates ABA biosynthesis, the impaired *OsMNT2* degradation would lead to increased ABA levels, improving tolerance to salt stress (Jiang et al., 2019). Zhang et al. reported that miR166 knock-down lines (STTM166), in which miR166 activity is suppressed using a Short Target Tandem Mimic construct designed to sequester and degrade the cognate miRNA (Yan et al., 2012), as well as lines overexpressing a miR166-resistant version of its major target gene (*OsHB4*/*OsHOX32*), exhibited a rolled-leaf phenotype, which is normally displayed by rice plants under drought stress (Zhang et al., 2018). The rolled-leaf phenotype, along with reduced stomatal conductance, transpiration rate and xylem vessels diameter, contributed to the increased survival rates of these transgenic lines under drought stress (Zhang et al., 2018). In an early microarray study, miR169g and miR393 were reported to be strongly upregulated and transiently induced by drought stress, respectively (Zhao et al., 2007). Interestingly, miR169g was more strongly induced in roots than in shoots, suggesting a potential role of this miRNA in the rapid response to drought stress in root tissues (Zhao et al., 2007). The negative regulation of drought tolerance by miR393 was further confirmed by overexpressing this miRNA, as plants exhibited increased tillers and early flowering, as well as reduced tolerance to drought stress (Xia et al., 2012). Similarly, miR535 has also been identified as a negative regulator of drought and salinity tolerance, as both STTM inhibition and CRISPR-Cas9-mediated knock-out of miR535 significantly increased seedling survival rate under drought and salinity stress, whereas overexpression of miR535 increased plant susceptibility to drought stress compared to the wild-type (WT) plants. Interestingly, the rice-specific miR820 was found to be downregulated under drought stress in mature panicles, along with its target gene (*OsDRM2*) (Jeong et al., 2011). These seemingly incoherent results could be explained by the existence of two distinct isoforms of miR820: the canonical 21-nt miR820.1, which is loaded into AGO1 protein and regulates *OsDRM2* expression through mRNA cleavage, and the 24-nt miR820.2, which is incorporated into AGO4 protein and directs DNA methylation around the target site within *OsDMR2* locus (Wu et al., 2010).

Three copper-responsive miRNA families (miR397, miR408 and miR528) have also been implicated in rice tolerance to drought stress (**Figure 1**; **Supplementary Table 1**). All three copper-responsive miRNA families, along with four other miRNA families, were upregulated under drought stress conditions in drought-tolerant varieties (Nagina (N22) and Vandana), but downregulated in drought-sensitive varieties (Pusa Basmati 1 (PB1) and IR64) (Mutum et al., 2013; Balyan et al., 2017). Interestingly, the absence of significant sequence variation in the osa-miR408 genomic loci among the four varieties suggests that the contrasting expression patterns likely arise from variety-specific variations in upstream signal transduction components rather than from cis-regulatory differences (Mutum et al., 2013), further highlighting the high level of complexity underlying miRNA-mediated stress responses. Notably, expression of *OsSPL9*, a transcription factor that regulates both miR408 and miR528, was induced during drought stress in both N22 and Vandana and reduced in both PB1 and IR64. As a result, both the transcription factor (*OsSPL9*) and its downstream target genes (miR408 and miR528) exhibited a variety-specific response to drought stress (Mutum et al., 2013; Balyan et al., 2017). Furthermore, for miR408, this phenomenon was restricted to drought stress and was not observed under other abiotic stresses, as both N22 and PB1 showed similar miR408 upregulation under heat, cold, and salt stress (Mutum et al., 2013). Additionally, in the drought-sensitive rice cultivar PB1 overexpressing miR408, increasing copper supply to plastocyanin led to an increase in electron transport rate, biomass, and ROS levels. These effects could be attributed to the miR408-mediated decrease of copper-containing protein targets, particularly members of the uclacyanin-like protein family, thereby enhancing the plant’s tolerance to drought stress (Balyan et al., 2023). Finally, co-overexpression of the three copper-responsive miRNAs in the *Nipponbare* variety, which simultaneously silenced several laccase target genes, resulted in improved tolerance to drought stress throughout the growth period, as evidenced by reduced leaf damage, lower lipid peroxidation, and higher survival rate (Hong et al., 2024).

### 4.2 Salt stress

With more than 40% of all agricultural land suffering from salinization and over 50% of all arable land expected to suffer from soil salinization by 2050 as a result of poor irrigation practices and climate changes (Parmar et al., 2020; Yuan et al., 2024), salt stress is one of the main environmental challenges impacting rice production. Rice is considered one of the most salt-sensitive crops (Faiyue et al., 2012) and its susceptibility to salinity stress varies depending on the developmental stage (Islam and Karim, 1970; Panda et al., 2016). To cope with salinity stress, plants activate a variety of acclimation mechanisms, including signaling cascades, ion transport regulation, and miRNA-mediated gene regulation (Das et al., 2021). Several studies reported a significant number of salt-responsive miRNAs in different plant species such as rice (Macovei and Tuteja, 2012; Mondal et al., 2018), maize (Ding et al., 2009; Fu et al., 2017), soybean (Dong et al., 2013), radish (Sun et al., 2015), *Populus euphratica* (Li et al., 2013), and *A. thaliana* (Liu et al., 2008).

A total of 15 miRNA families have been reported as downregulated by salt stress, while 12 were upregulated (**Figure 1**; **Supplementary Table 1**). As such, miR168a-5p expression was upregulated in both roots and shoots of salt-stressed rice plants, followed by downregulation of its target genes *OsAGO1* and *OsNPF2.4*, resulting in decreased germination rate, seedling growth and salt tolerance compared to WT plants (Xia et al., 2023). On the other hand, miR168 knock-down lines (STTM168) exhibited improved salt tolerance and increased plant height and root length compared to WT plants, indicating a potential role for the miR168/AGO1 regulatory module in the salt stress response (Wan et al., 2022). miR169g and miR169n were identified as high salinity-promoted miRNAs, while the expression of one of the miR169 family target genes, *OsNF-YA2*, which regulates plant cell elongation and carbohydrate metabolism, was downregulated (Zhao et al., 2009a). Overexpression of miR171c significantly decreased salt tolerance at both germination and seedling stages, reduced proline accumulation, and increased water loss rate, suggesting its role as a negative regulator of salt stress tolerance (Yang et al., 2017). Likewise, miR393 has been identified as a negative regulator of salt tolerance: miR393-overexpressing plants exhibited increased tillering and early flowering, as well as reduced tolerance to salt stress and hypersensitivity to auxin (Gao et al., 2011; Xia et al., 2012).

Salt stress also repressed miR396c, whose overexpressing lines showed reduced salt stress tolerance compared to WT plants (Gao et al., 2010). In line with this, Yuan et al. reported increased survival rate and growth potential in transgenic lines overexpressing the target mimicry of miR396b (MIM396) or its target gene *OsGRF6* under salt stress, together with reduced H_2_O_2_ accumulation and improved ROS scavenging (Yuan et al., 2024). *OsZNF9*, a negative regulator of rice salt tolerance, directly binds to the promoter of miR396b and modulates the expression of the miR396b/*OsGRF6* module, thereby regulating salt tolerance response and grain yield. *OsMYB3R*, on the other hand, was identified as a downstream target of miR396b/*OsGRF6*, and its overexpression significantly enhanced salt tolerance by maintaining ROS homeostasis (Yuan et al., 2024).

Improved ROS scavenging was also observed after overexpression of miR528, along with increased ascorbic acid (AsA) and ABA content, due to the downregulation of its target gene L-ascorbate oxidase (*OsAAO2*) (Wang et al., 2021) (**Figure 1**; **Supplementary Table 1**). Conversely, silencing miR528 or overexpressing *OsAAO2* decreased AsA content, an important plant antioxidant that, among other functions, promotes ABA biosynthesis and reduces ROS levels (Wang et al., 2021). Consistently, knock-out of *OsRFI2*, another miR528 target encoding a RING-type E3 ubiquitin ligase, conferred high salt tolerance, whereas its overexpression increased rice sensitivity to salt stress (Zhao et al., 2025). Indeed, *OsRFI2* degradation induced by miR528 limits OsRFI2 proteins transported into the chloroplast, reducing ubiquitination and degradation of OsAPX8 and promoting the conversion of H_2_O_2_ to H_2_O and O_2_ (Zhao et al., 2025).

Finally, the rice-specific miRNA miR820, which regulates the *de novo* methyltransferase *OsDRM2*, was found to be upregulated in leaf tissues and downregulated in roots under salt stress, as well as under drought and high temperature stress (Sharma et al., 2015b) (**Figure 1**; **Supplementary Table 1**). These results may suggest a crucial role for the miR820/*OsDRM2* module in genome defense by reducing transposons and epigenetic activity during reproductive development and stress (Cao and Jacobsen, 2002; Sahu et al., 2013). In a later study, Sharma et al. reported that plants overexpressing the canonical 21-nt miR820.1 exhibited enhanced plant architecture and grain yield under long-term salt stress conditions, as well as better water use efficiency and higher proline accumulation (Sharma et al., 2021). However, like their WT counterparts, miR820-overexpressing plants could tolerate mild salinity conditions (50–200 mM) but could not withstand high salt concentrations (200–300 mM) (Sharma et al., 2021).

### 4.3 Temperature stress

Due to geographical locations and seasonal variations, plants often face extreme temperatures (i.e. cold and heat stress) which negatively affect plant growth and productivity (Raza et al., 2023). In rice, the impact of harsh temperature conditions on plant development differs depending on the developmental stage: cold stress has a greater impact at the germination and reproductive anthesis stages, whereas heat stress has more severe effects at the reproductive anthesis and seed ripening stages (Jeong and Green, 2013). Cold- or heat-responsive miRNAs have been identified in various plant species such as rice (Lv et al., 2010; Mangrauthia et al., 2017), wheat (Song et al., 2017), *Brassica rapa* (Zeng et al., 2018), *A. thaliana* (Navarro et al., 2006; Samuel et al., 2008), *Betula luminifera* (Pan et al., 2017a, Pan et al., 2017b), *Saccharina japonica* (Liu et al., 2015a; Liu et al., 2015c) and *Medicago sativa* (Shu et al., 2016).

#### 4.3.1 Cold

In an early microarray study of rice seedlings, Lv et al. identified 14 cold-responsive miRNA families, of which 10 were downregulated and four were upregulated (**Figure 1**; **Supplementary Table 1**) (Lv et al., 2010). Interestingly, phytohormone regulatory elements were identified in the upstream regions of most cold-responsive miRNAs, suggesting an important role for hormones in the miRNA-mediated cold defense system (Lv et al., 2010). Comparative analysis of the miRNA profiles of rice anthers between a cold-sensitive (Sasanishiki) and a cold-tolerant (Hitomebore) rice cultivar revealed that cold stress induced the expression of four miRNAs (miR398b, miR531a/c, miR1432-5p, and miR5072) in the former, and five miRNAs (miR397b, miR398b, miR408-3p, miR528-5p, and miR1432-5p) in the latter (Maeda et al., 2016). In particular, the downregulation of *SOD* genes in the cold-tolerant variety, which are targets of the upregulated miR398b and miR528-5p, may contribute to the maintenance of normal tapetum degradation and subsequent pollen maturation under cold stress (Maeda et al., 2016).

Cold tolerance positive regulators include miR319, miR1320 and, as for drought, the copper-responsive miR397, miR408 and miR528 (**Figure 1**; **Supplementary Table 1**). Overexpression of miR319 or downregulation of its target genes (*OsPCF5*, *OsPCF6*, *OsPCF8* and *OsTCP21*) resulted in lower accumulation of ROS and greater accumulation of free proline, which facilitates osmoregulation and protects plants from dehydration due to cold stress (Yang et al., 2013; Wang et al., 2014b). The rice-specific miR1320, which targets an APETALA2/ethylene-responsive factor transcription factor (*OsERF096*) and naturally shows higher expression levels at the early vegetative stage, was found to be downregulated under cold stress (Sun et al., 2022). Genetic evidence showed that rice cold tolerance could be improved by miR1320 overexpression and reduced by miR1320 knock-down through regulation of its target gene *OsERF096*, which represses jasmonic acid (JA) biosynthesis and JA-mediated cold signaling pathway as well as ROS scavenging (Sun et al., 2022). Furthermore, *OsPHD17*, another target gene of miR1320, also acts as a negative regulator of cold tolerance in rice seedlings by modulating ROS homeostasis, flavonoid accumulation, and JA-mediated signaling pathway under cold stress (Wang et al., 2024). Overexpression of *OsPHD17* reduced, whereas knock-down of *OsPHD17* increased, cold tolerance of rice seedlings, partly via the CBF-mediated cold signaling pathway (Wang et al., 2024).

Regarding the copper-responsive miRNAs, overexpression of miR408, followed by the repression of its targets, the uclacyanin-like protein (*OsUCL31*) and an auxin-responsive gene (*OsIAA6*), resulted in enhanced tolerance to cold stress at both early and young seedling stages (Sun et al., 2018b). Similarly, overexpression of the monocot-specific miR528 improved cold tolerance by increasing cell viability and growth as well as antioxidant enzyme activity (Tang and Thompson, 2019). It has been suggested that such enhanced cold tolerance could result from miR528-mediated downregulation of *OsD3*, a positive regulator of *OsMYB30*, thereby decreasing *OsMYB30* levels, inducing β-amylase (*BMY*) gene expression, and increasing sugar content (Tang and Thompson, 2019). Furthermore, co-overexpression of the three copper-miRNAs, which simultaneously silenced several laccase target genes, resulted in reduced lignin content, increased flavonoid accumulation and significantly enhanced tolerance to cold stress throughout the growth period, resulting in higher pollen fertility and setting rate (Hong et al., 2024).

Conversely, miR156k, miR535, miR1425 and miR1432 are known negative regulators of the cold stress response (**Figure 1**; **Supplementary Table 1**). In seedlings, Cui et al. showed that overexpression of miR156k reduced tolerance to cold stress, as evidenced by lower survival rates, chlorophyll content, proline content and weaker ROS scavenging capacity (Cui et al., 2015). In addition to its negative role in drought and salinity tolerance, miR535 overexpression under cold stress suppresses early seedlings growth, aggravating cold-induced cell death, decreasing the activity of ROS-scavenging enzymes, and affecting osmotic regulation (Sun et al., 2020). Overexpression of miR535 also reduced the levels of core components of the CBF-mediated cold signaling pathway (Sun et al., 2020). Small RNA sequencing analysis of rice plants revealed that miR1425 was downregulated under cold stress conditions (Jeong et al., 2011) and that the consequent upregulation of its target gene, *OsRF-1*, a fertility restorer, confers cold tolerance at the booting stage by increasing the number of potentially fertile pollen grains (Komori and Imaseki, 2005). Finally, overexpression of the monocot-specific miR1432 or suppression of its target gene (*OsACA6*) resulted in reduced cold tolerance (Dai et al., 2024). Furthermore, rice plants overexpressing miR1432 exhibited weakened vigor, dwarfism, yellowed leaves and reduced fertility, indicating an important role of the miR1432/*OsACA6* module in plant growth and development (Dai et al., 2024).

#### 4.3.2 Heat

In a genome-wide study, Li et al. identified 39 heat-responsive miRNAs in rice panicles, 24 of which were upregulated and 15 were downregulated (**Figure 1**; **Supplementary Table 1**) (Li et al., 2015). Similarly, Sailaja et al., reported that nine miRNAs were differentially expressed in roots and/or shoots of rice seedlings under heat stress (**Figure 1**; **Supplementary Table 1**) (Sailaja et al., 2014). Among these, four miRNAs (miR156, miR162, miR167 and miR398) were consistently downregulated in both tissues, while three miRNAs (miR160, miR169 and miR1884) exhibited opposite expression patterns, being upregulated in shoots and downregulated in roots (Sailaja et al., 2014). Comparative analysis of miRNA profiles between a heat-sensitive (Vandana) and a heat-tolerant (Nagina 22) cultivar revealed that miR1436, miR5076, miR5161 and miR6253 were differentially expressed in stressed root and shoot tissues of both genotypes, suggesting a role in heat stress responses (Mangrauthia et al., 2017). In addition, differential expression of miR1439, miR2096, miR2106, miR2875, miR3981, miR5079, miR5151, miR5484, miR5792 and miR5812 was observed only under high temperature stress in the heat-tolerant cultivar, suggesting a specific role of these miRNAs to heat stress tolerance (Mangrauthia et al., 2017). Finally, an integrated analysis combining genome-wide miRNA profiling with QTL mapping at the flowering stage in both a heat-tolerant (Gan-Xiang-Nuo (GXN)) and a heat-sensitive (Hua-Jing-Xian-74 (HJX)) cultivar identified 102 differentially expressed genes under heat stress (Liu et al., 2017d). Among these, four miRNAs (miR164d, miR166i-3p, miR168a-3p and miR397b) were consistently downregulated in both cultivars after heat stress (Liu et al., 2017d), suggesting a variety-independent regulatory role in heat tolerance. On the other hand, five miRNAs (miR159a.1, miR159b, miR396e-5p, miR396f-5p and miR528-3p) showed opposite expression patterns in GXN and HJX, indicating a variety-specific response to heat stress. Furthermore, the role of miR169r-5p in heat tolerance was functionally confirmed, as its overexpression in the heat-sensitive variety *Zhonghua11* (ZH11) resulted in higher spikelet fertility (55.79%) compared to WT plants (42.50%) (Liu et al., 2017d). Overall, these studies indicate that heat-responsive miRNAs in rice operate through both conserved regulatory pathways shared across cultivars or tissues and highly genotype- or tissue-specific networks, highlighting the importance of considering both tissue context and variety-specific signaling pathways when interpreting miRNAs function under heat stress.

### 4.4 Heavy metal stress

Heavy metals can be classified into two groups: essential heavy metals such as copper (Cu), iron (Fe), zinc (Zn), and manganese (Mn), which play central roles in enzymatic and biochemical reactions in plant cells at low concentrations but become toxic at higher concentrations, and non-essential heavy metals such as cadmium (Cd), chromium (Cr), aluminum (Al), arsenic (As) and mercury (Hg), which are toxic even at low concentrations (Rascio and Navari-Izzo, 2011; Gielen et al., 2012; Gupta et al., 2014). Although heavy metals occur naturally in the Earth’s crust due to geological processes (weathering of rocks and volcanic activity), anthropogenic activities (e.g. waste incineration, wastewater treatment and mining) have led to a significant increase in contamination of water and soil, threatening both agriculture and food safety (Raza et al., 2023). Rice grains grown in fields contaminated with heavy metals represent one of the main sources of human uptake of these elements (Arif et al., 2019). To counteract and mitigate the harmful effects of heavy metal uptake and accumulation, plants employ vacuolar compartmentalization, chelation, sequestration and exclusion (Das et al., 2021), processes that have been shown to be miRNA-dependent in various plant species, including rice (Ding et al., 2011), *M. truncatula* (Zhou et al., 2008b), *Brassica napus* (Huang et al., 2010), and *A. thaliana* (Ding and Zhu, 2009).

#### 4.4.1 Cadmium

Considered the most toxic heavy metal, cadmium interferes with the uptake, transport and utilization of essential nutrients and water, reduces photosynthesis and alters enzyme activities (Gallego et al., 2012). In an early microarray study, Ding et al. identified ten Cd-responsive miRNA families, of which only one (miR528) was upregulated and the remaining nine (miR156, miR162, miR166, miR168, miR171, miR390, miR396, miR444 and miR1432) were downregulated (Ding et al., 2011).

These results were further validated by several miRNA-specific studies, which confirmed the differential expression patterns of these miRNAs under Cd stress. For example, miR166 is repressed in rice seedling roots by Cd stress, leading to the upregulation of its target gene *OsHB4*, a class III HD-Zip protein likely involved in the regulation of genes responsible for Cd uptake and translocation (Ding et al., 2018). Moreover, overexpression of miR166 or silencing of *OsHB4* improved rice tolerance to Cd stress by reducing Cd-induced oxidative stress, Cd translocation from root to shoot, and Cd accumulation in the grains, whereas overexpression of *OsHB4* triggered an opposite effect (Ding et al., 2018). Cadmium-promoted miR390 downregulation in rice seedling roots leads to the upregulation of the stress-responsive LRR-like kinase *OsSRK* (Ding et al., 2016). Transgenic plants overexpressing miR390 displayed higher Cd accumulation, oxidative stress and delayed seedling growth compared to WT plants (Ding et al., 2016).

In contrast, the upregulation of the monocot-specific miR528 after Cd stress likely modulates Cd absorption via the downregulation of its target gene *OsUCL23* and associated effects on ROS signaling (Tan et al., 2025). Interestingly, the transcription factor WRKY51 has been demonstrated to bind to the promoters of *MIR528* and *OsUCL23*, contributing to Cd responses and promoting ROS accumulation in a dual regulatory manner: it activates *OsUCL23* expression and inhibits the expression of its post-transcriptional regulator miR528. Indeed, transgenic lines silencing or overexpressing miR528 have been reported to exhibit increased and decreased ROS levels, respectively, and contrasting sensitivity to Cd stress compared to WT plants (Tan et al., 2025). An opposite trend was observed upon overexpression or silencing of *OsUCL23* (Tan et al., 2025).

Interestingly, miR535 expression pattern was found to depend on the Cd concentrations, being upregulated at low Cd (2 μM) and downregulated at high Cd (200 μM) levels (Yue et al., 2024). Downregulation of the miR535 target gene *OsSPL7* decreased its binding affinity to the promoter of *OsNramp5*, a key Cd transporter gene, thereby increasing *OsNramp5* transcript levels to mediate Cd absorption. Overexpression of miR535 resulted in enhanced *OsNramp5* expression, Cd translocation and root length, whereas knock-down of miR535 or overexpression of *OsSPL7* resulted in reduced *OsNramp5* expression, Cd translocation and root length under cadmium stress conditions (Yue et al., 2024).

#### 4.4.2 Arsenic

Compared to other cereals such as wheat and barley, rice is highly efficient in the uptake and accumulation of arsenic, especially the more toxic inorganic forms arsenate [As(V)] and arsenite [As(III)] (Su et al., 2010), which makes arsenic contamination a major concern for the human population worldwide. As(V) is the main arsenic species in aerobic soils. It is taken up by roots via phosphate transporters and can substitute inorganic phosphate in many biological processes. As(III) is predominant in anaerobic environments such as submerged paddy soils, and can react with cysteine sulfhydryl groups resulting in loss of function of enzymes and proteins (Zhao et al., 2009b).

Using contrasting As-accumulating rice cultivars, Sharma et al. found that arsenic exposure modulated a large number of miRNA families in an As(III) and As(V)-specific manner (**Figure 1**; **Supplementary Table 1**) (Sharma et al., 2015a). In response to As(III), 30 and 62 miRNA families were modulated in the High As-accumulating Rice Germplasm (HARG) and Low As-accumulating Rice Germplasm (LARG), respectively, whereas As(V) exposure affected 81 and 77 families in HARG and LARG (Sharma et al., 2015a). Although many miRNAs were regulated in a cultivar-specific way, 14 miRNAs were consistently modulated by both As(III) and As(V) in both cultivars (Sharma et al., 2015a). Among these, members of the miR396, miR399, miR408, miR528, miR1861, miR2102, and miR2907 families were predominantly upregulated, whereas members of the miR164, miR171, miR395, miR529, miR820, miR1432, and miR1846 families were mainly downregulated (Sharma et al., 2015a).

In an early genome-wide study, Liu and Zhang identified 67 As(III)-responsive miRNAs in rice seedling roots, with 19 and seven miRNA families significantly down- and upregulated, respectively (**Figure 1**; **Supplementary Table 1**) (Liu and Zhang, 2012). In particular, upregulation of miR408 and miR528 followed by downregulation of their copper-containing target genes might limit copper usage for essential biological processes under stress conditions. Liu et al. further showed that transgenic plants overexpressing miR528 were more sensitive to As(III) compared to WT plants, likely due to a strong alteration in antioxidant enzyme activity and amino acid profiles, as well as impaired As(III) uptake, translocation and tolerance systems (Liu et al., 2015b). In particular, the expression of four genes (*OsLPR4*, *OsLPR5*, *OsLAC2*, and *OsLAC6*) involved in oxidative stress responses showed a negative correlation with miR528 in response to As(III) treatment (Liu et al., 2015b). However, whether these genes are direct or indirect targets of miR528 remains to be investigated. Notably, the study by Liu and Zhang also provided the first evidence for exonic miR6256, which is constitutively expressed in roots, leaves, seeds and reproductive tissues, and was strongly downregulated after arsenite stress (Liu and Zhang, 2012). miR6256 was predicted to target its host gene *Os2ODD2*, a 2-oxoglutarate-dependent dioxygenase, a class of enzymes implicated in redox homeostasis and stress-related metabolic pathways (Farrow and Facchini, 2014). A similar miRNA-host gene regulation has been reported for exonic miR3981 (Li et al., 2011), suggesting a potentially conserved mechanism in which exonic miRNAs fine-tune host gene expression in response to environmental stress through DCL-mediated miRNA regulation.

Further evidence for the differential regulation promoted by As(III) came from a global transcriptome analysis, in which Yu et al. identified 22 miRNA families in roots and 30 miRNA families in shoots responding to As(III) treatment (**Figure 1**; **Supplementary Table 1**) (Yu et al., 2012). Interestingly, the downregulation of miR156j, which is known to target *SPL* transcription factors involved in the control of plant development, was later on found to be more pronounced in root tissue and during the seedling developmental stage, followed by flowering and tillering (Pandey et al., 2020). Finally, Liu reported that two miRNAs (miR6247 and miR6249) were downregulated after As(III) treatment, whereas six miRNAs (miR1440b, miR6246, miR6250, miR6252, miR6253 and miR6255) were significantly upregulated (Liu, 2012).

#### 4.4.3 Chromium

Although chromium, particularly its hexavalent form [Cr(VI)], is considered one of the most abundant and harmful heavy metals in water and soil worldwide, only one study to date has identified Cr-responsive miRNAs in rice plants exposed to short- or long-term Cr stress (Dubey et al., 2020). Under short-term Cr exposure, six miRNAs (miR156, miR159, miR396, miR397, miR2877 and miR5072) were upregulated and six miRNAs (miR160, miR169, miR171, miR408, miR444 and miR1883) were downregulated (**Figure 1**; **Supplementary Table 1**). In contrast, long-term Cr exposure led to the upregulation of only two miRNAs (miR166 and miR171) and the downregulation of three miRNAs (miR396, miR444 and miR5072) (Dubey et al., 2020). Interestingly, miR171, miR396 and miR5072 showed differential expression depending on the stress duration, while miR444 was the only miRNA that was consistently downregulated at both time points (Dubey et al., 2020). Notably, miR396 differential expression under short and long-term Cr exposure may indicate that the plant possesses both acute and prolonged defense mechanisms, as miR396 target genes, ATP-binding cassette (*ABC*) transporters and growth-regulating factors (*GRFs*), are primarily involved in heavy metal detoxification and overall plant development (Dubey et al., 2020). Finally, under short-term Cr stress, downregulation of miR160 and upregulation of miR159, followed by inverse expression patterns of their target genes, auxin response factors (*ARFs*) and *MAPK* signaling components, respectively, might increase auxin content, which may be required to counteract Cr stress (Dubey et al., 2020). Overall, these results suggest that rice relies on distinct miRNA-mediated regulatory pathways to cope with either acute or chronic Cr stress, with early responses involving rapid detoxification mechanisms and long-term exposure focusing on the maintenance of growth and development.

#### 4.4.4 Aluminum

Although aluminum toxicity has been recognized as a major limiting factor for plant productivity in acidic soils, only two studies to date have investigated the expression of Al-responsive miRNAs in rice (Lima et al., 2011; Xu et al., 2017) (**Figure 1**; **Supplementary Table 1**). Lima et al. reported that 12 miRNA families were downregulated in rice roots under aluminum stress, while six miRNA families were upregulated (Lima et al., 2011). Among these, reduced miR395 expression, together with increased expression of key proteins involved in sulfur homeostasis in response to aluminum stress, supports the findings of Yang et al, in which genes related to sulfur metabolism in roots were induced after Al treatment (Yang et al., 2007). Downregulation of miR398, followed by upregulation of its *SOD* target genes, important enzymes involved in ROS scavenging, also supports the increased activity of SOD in seedlings treated with high Al concentrations (Sharma and Dubey, 2007). Finally, upregulation of miR160, miR166 and miR528, and downregulation of miR393 might contribute to the fine-tuning of root growth responses after Al treatment (Lima et al., 2011).

In contrast, Xu et al. reported different expression patterns for miR528 and miR160a under aluminum stress (Xu et al., 2017). In their study, Al-treated rice seeds showed increased expression of miR159a and decreased expression of miR160a, miR398a, and miR528, followed by a negative correlation with their target genes (*OsGAMYB*, *OsARF10*, *OsSODCC2*, and *OsAAO2*, respectively) (Xu et al., 2017). Additionally, pre-treatment of the rice seeds with H_2_ (0.39 mmol L^-1^) further reduced the transcript level of miR398a, while reversing the transcription levels of miR159a, miR160a, and miR528. As a result, upregulation of *OsSODCC2* might improve the resistance of seeds to oxidative stress caused by Al stress, whereas the combined downregulation of *OsARF10* and upregulation of *OsGAMYB* might alleviate germination inhibition caused by Al stress (Xu et al., 2017).

### 4.5 Nutrient homeostasis

Nutrients are essential for plant growth, development and yield and can be classified into two groups: macronutrients such as nitrogen (N), phosphorus (P), potassium (K) and sulfur (S) which are required for optimal plant growth and development, and micronutrients such as iron (Fe), copper (Cu) and zinc (Zn) which function mainly as cofactors of metabolic enzymes and protein complexes in the electron transport chain (Shriram et al., 2016). Nutrients acquisition, assimilation and metabolism are tightly regulated in plants and their limitation can have drastic effects on the optimal plant growth and productivity (Shriram et al., 2016). Indeed, miRNA-dependent nutrient uptake and transport has been extensively investigated in different species (Pant et al., 2009; Lundmark et al., 2010; Hu et al., 2011; Jeong et al., 2011; Liang et al., 2012; Nischal et al., 2012; Zhao et al., 2012; Zhao et al., 2013; Hackenberg et al., 2013; Pei et al., 2013).

#### 4.5.1 Nitrogen

Nitrogen is an essential macronutrient for plant growth and development, being an essential component of enzymes, proteins, hormones, nucleic acids, alkaloids, vitamins and secondary metabolites (Shrestha et al., 2020). Plants obtain nitrogen from the soil mainly in the form of nitrate (NO_3_^-^) (Yan et al., 2014). Given the high nitrogen requirements of major food crops, nitrogen represents a serious limiting factor for agricultural productivity (Yuan et al., 2015). However, less than half of the nitrogen fertilizer applied is actually utilized by the plants, and excessive use contributes to global warming through nitrous oxide emissions and water pollution from nitrate leaching (Nischal et al., 2012).

The comparison between a low-N sensitive and a low-N tolerant rice cultivar, revealed that 15 miRNA families were differentially expressed in both genotypes under N limitation (Nischal et al., 2012) (**Figure 1**; **Supplementary Table 1**). The downregulation of miRNAs expression was more pronounced in the low-N tolerant variety than in the low-N sensitive variety, suggesting the activation of specific target genes in response to nitrogen starvation in the former genotype (Nischal et al., 2012). For instance, N deficiency repressed miR168a-5p, resulting in increased expression of the nitrogen transporter *OsNPF2.4*, which promoted long-distance nitrate transport between roots and shoots (Xia et al., 2023). Conversely, high N supply promoted miR168a-5p, repressing the expression of *OsNPF2.4* and attenuating N root-to-shoot mobilization. Indeed, total N content was significantly lower in the leaves of transgenic lines overexpressing miR168a-5p compared to WT plants (Xia et al., 2023).

Similarly, miR169o is downregulated by N limitation, resulting in the direct upregulation of two subunit A genes of nuclear factor Y (*OsNF-YA1* and *OsNF-YA4*), involved in development and abiotic stress response in plants (Yu et al., 2018). *NF-YAs* mediate the control of nitrate transporter genes (*OsNRT1* and *OsNRT2*) under N-deficient conditions, thus impacting nitrogen use efficiency (NUE) (Yu et al., 2018). In line with this, overexpression of miR169o attenuated the N starvation symptoms, resulting in taller plants that accumulated more biomass, with increased nitrate and total amino acid contents in the roots (Yu et al., 2018). Similarly, overexpression of the monocot-specific miR528 in creeping bentgrass (*Agrostis stolonifera*) improved plant tolerance to N limitation by increasing biomass accumulation, chlorophyll content, photosynthetic capability, total N accumulation and nitrite reductase activity, while decreasing ascorbic acid oxidase (*OsAAO2*) activity (Yuan et al., 2015). Knock-down of miR396e, f resulted in a similar trend under N deficiency (Zhang et al., 2020b). The resulting upregulation of its targets, the growth-regulating factors *OsGRF4*, *OsGRF6* and *OsGRF8*, improved photosynthesis, grain size, panicle branching, N content and aboveground biomass, leading to a 15% increase in grain yield and more efficient NUE, as evidenced by increased expression of genes involved in nitrogen assimilation and utilization (Zhang et al., 2020b).

On the other hand, the monocot-specific miR444 acts as a negative regulator of tolerance to N starvation by repressing the MADS-box transcription factors (*OsMADS23*, *OsMADS27* and *OsMADS57*) involved in the regulation of nitrate and auxin signaling pathways in roots (Pachamuthu et al., 2022) (**Figure 1**; **Supplementary Table 1**). Transgenic rice plants expressing a miR444 target mimic or overexpressing a miR444-resistant *OsMADS27* mutant gene showed improved root development and tolerance to abiotic stress (Pachamuthu et al., 2022). Furthermore, overexpression of miR444a reduced the remobilization of nitrate from old to young leaves, thereby increasing rice sensitivity to N-limiting conditions, and altered the architecture of primary and adventitious roots in response to different nitrate concentrations (Yan et al., 2014). Interestingly, miR444a was also significantly upregulated under phosphate (Pi) starvation conditions and miR444a overexpressing plants showed altered root architecture, increased Pi accumulation and expression of Pi transporter genes under Pi-starvation conditions (Yan et al., 2014). Taken together, these results suggest the miR444a/*OsMADS27* module is involved in multiple nutrient deficiency signals in rice, potentially acting as a central regulatory node coordinating root architecture adjustments under different nutrient limitation conditions.

High N levels promote rice tillering via upregulation of miR393, followed by upregulation of two auxin receptors, *OsAFB2* and *OsTB1*, which results in reduced sensitivity to auxin in axillary buds and stabilized *OsIAA6* (Li et al., 2016a). This regulatory network, and the N-promoted tillering, were reversed in the CRISPR-Cas9-mediated miR393 knock-out line, mimicking the effects of N limitation (Li et al., 2016a).

#### 4.5.2 Phosphorus

Phosphorus is an essential macronutrient in plants, and a building block for molecules such as nucleotides, co-enzymes, ATP, nucleic acids and phospholipids (Hu et al., 2015). However, the availability of inorganic phosphate (Pi) in soils is often limited, resulting in phosphorus starvation conditions (Hu et al., 2015). Therefore, plants have evolved specialized mechanisms, including morphological, physiological, and biochemical responses, to adapt and enhance Pi acquisition and availability (Raghothama, 1999).

Two miRNAs were found to be upregulated under P starvation in rice: miR399 and miR827, both controlled by *OsPHR2* (homolog to *AtPHR1* in *A. thaliana*) under Pi starvation (Bari et al., 2006; Zhou et al., 2008a; Lin et al., 2010; Wang et al., 2012) (**Figure 1**; **Supplementary Table 1**). miR399 downregulates the expression of *OsLTN1* (*AtPHO2* homolog), a negative regulator of Pi uptake and translocation (Hu et al., 2011). Overexpression of miR399 under normal Pi conditions caused overaccumulation of this nutrient in rice leaves and symptoms of Pi toxicity (Hu et al., 2011). Interestingly, induction of miR399 expression was also observed under Fe, K, Na and Ca starvation, suggesting the involvement of miR399 in multiple nutrient starvation responses (Hu et al., 2015). Transgenic plants overexpressing miR399 and *OsLNT1* loss-of-function mutants also showed an increase in the concentrations of Fe, K, Na and Ca due to the upregulation of a number of genes involved in nutrient absorption at the root level, in addition to their involvement in Pi starvation (Hu et al., 2015). Regarding miR827, it targets two SPX-MFS genes (*OsSPX-MFS1* and *OsSPX-MFS2*), which are thought to be implicated in Pi sensing or transport. Interestingly, the two target genes show opposite expression patterns in response to Pi starvation: while *OsSPX-MFS1* is downregulated, *OsSPX-MFS2* is significantly upregulated; suggesting a more complex miRNA/target regulation loop (Lin et al., 2010). Overexpression of miR827 or mutation of *OsSPX-MFS1* under Pi starvation conditions resulted in impaired Pi homeostasis in leaves and Pi overaccumulation in older leaves, which was associated with altered responses of some phosphate starvation-induced genes (Wang et al., 2012).

#### 4.5.3 Sulfur

Sulfur, which occurs in nature mainly as sulfate (SO_4_^2-^), is an essential component for the synthesis of amino acids (especially cysteine and methionine), vitamins, and lipids, playing a key role in cellular energy homeostasis (Hu et al., 2015). Although sulfur deficiency in the soils was rare in the past, it has become progressively more common in recent years due to increasing nutrient depletion associated with intensive cropping and reduced application of S-containing fertilizers (Pariasca-Tanaka et al., 2020).

In rice, miR395 was found to be significantly upregulated under sulfate starvation, resulting in the downregulation of its target genes (*OsAPS1*, *OsSULTR2;1* and *OsSULTR2;2*), which are involved in sulfate assimilation, translocation and accumulation (Jeong et al., 2011; Yang et al., 2022) (**Figure 1**; **Supplementary Table 1**). Rice, *A. thaliana* and tobacco plants overexpressing miR395b accumulated high levels of sulfate due to dysfunction of ATP sulfurylases and SULTRs, resulting in sulfate overaccumulation in leaves and impaired sulfate translocation from old to young leaves in S-sufficient conditions (Yang et al., 2022). Interestingly, because morphological abnormalities were observed in the latter two species but not in rice miR395b-OE lines, it has been proposed that other sulfur-related pathways, such as thiosulfate, hydrogen sulfite or sulfur dioxide assimilation pathways, may play a complementary role in rice, suggesting the existence of species-specific metabolic networks (Yang et al., 2022).

#### 4.5.4 Zinc

Zinc is an essential micronutrient in plants as it is an essential component of several enzyme systems (e.g. dehydrogenases, proteases, peptidases, alcohol dehydrogenase and superoxide dismutase), and many Zn-finger domain-containing proteins involved in transcriptional regulation (Broadley et al., 2007; Shrestha et al., 2020). Although Zn deficiency is one of the most widespread soil limitations affecting rice productivity, as the reduced function of alcohol dehydrogenase decreases the ability of rice seedlings to cope with anaerobic soil conditions (Moore and Patrick, 1988), only one study to date has investigated the expression of Zn-responsive miRNAs. Transcriptome analysis of miRNA profiles under Zn deficiency and/or Zn resupply identified 68 differentially expressed miRNAs (Zeng et al., 2019) (**Figure 1**; **Supplementary Table 1**). Among these, 23 miRNAs were upregulated under Zn deficiency (12 in shoots and 11 in roots), while 14 were downregulated (2 in shoots and 12 in roots) (Zeng et al., 2019). Interestingly, none of these miRNAs were responsive to Zn deficiency in both tissues, suggesting distinct miRNA-mediated regulatory networks between roots and shoots (Zeng et al., 2019). For example, upregulation of miR398 in shoots and miR528 in roots by Zn deficiency, followed by downregulation of their target genes (*OsCSDs* and *OsCCS* for miR398, and *OsAAO2* and *OsSOD4* for miR528), suggests their role in balancing ROS accumulation and scavenging under Zn stress (Zeng et al., 2019). In particular, downregulation of Zn-containing SODs, which can be functionally replaced by Fe- or Mn-containing SODs, further allows the allocation of limited Zn to other essential Zn-containing proteins to adapt to the low Zn environment (Zeng et al., 2019). Interestingly induction of miR397 in root and miR408 in shoot by Zn resupply suggests involvement in Cu-Zn nutritional interaction, as both miRNAs are also induced by Cu deficiency and their target genes encode various Cu-containing proteins (Pilon, 2017; Zeng et al., 2019).

## 5 miRNA-mediated responses to ionizing radiation in rice

Ionizing radiation (IR), defined as high-energy particles or waves capable of ionizing atoms and molecules, is a natural and evolutionary ancient stress factor whose intensity is increasing in the present biosphere as a result of anthropogenic activities. Atmospheric contamination from global fallout following nuclear weapons use and testing (e.g. Hiroshima and Nagasaki, Japan 1945; Bikini Atoll, Marshall Islands 1954) and severe nuclear accidents (e.g. Kysthym, Russia 1957; Chornobyl, Ukraine 1986; Fukushima, Japan 2011), has had substantial impacts on the global environment as well as on individual organisms and populations (Prăvălie, 2014; Beresford et al., 2016; Fesenko, 2019; Hughes et al., 2019; Ludovici et al., 2022). Because some radionuclides have long half-lives, nuclear contamination can persist for centuries, resulting in chronic exposure of local species across generations (Duarte et al., 2023). For sessile organisms like plants, it is therefore critical to understand the mechanisms governing IR impact on development, yield, and ultimately population dynamics.

**Figure 3:**
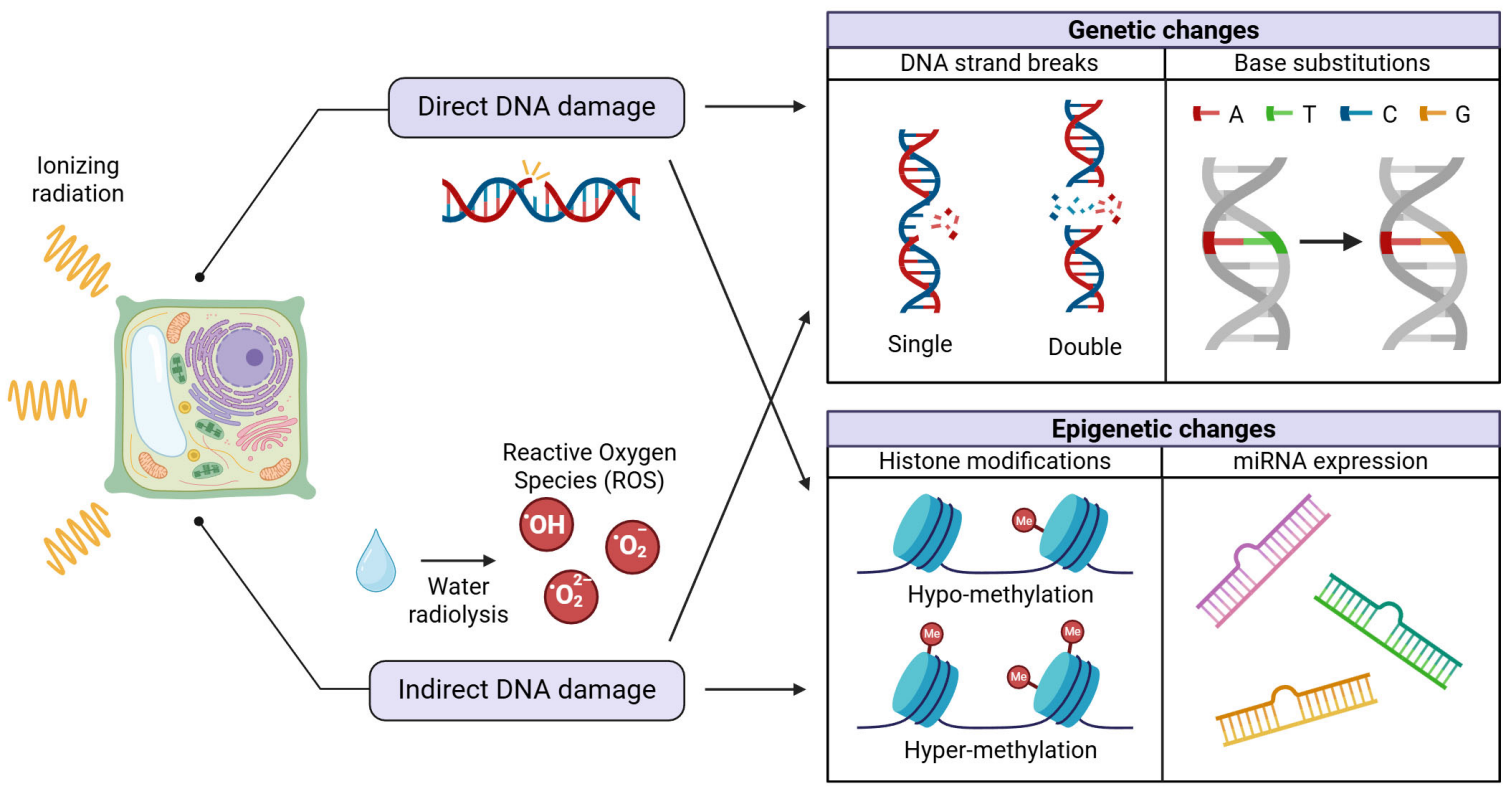
Cellular effects of ionizing radiation (IR). Ionizing radiation causes cellular damage through direct and indirect mechanisms. Direct DNA damage results from the interaction of ionizing radiation with the DNA molecule. Indirect DNA damage is caused by the generation of reactive oxygen species (ROS), which are products of water hydrolysis caused by the high-energy particles. Both pathways can lead to genetic changes (e.g., single- and double-strand breaks, base substitutions) and epigenetic modifications (e.g., histone modifications, miRNA differential expression). Created in BioRender. Bordignon, S. (2026) https://BioRender.com/rw5jnyx.

At the subcellular level, IR can cause a wide range of detrimental effects such as DNA damage (e.g., base substitutions, single- or double-strand breaks) and oxidative stress via ROS generation (**Figure 3**) (Duarte et al., 2023). Post-transcriptional regulations, such as those mediated by miRNAs, have been suggested to contribute to fast acclimation stress responses (Morad‐Talab and Hajiboland, 2016). In mammalian systems, miRNA responses to genotoxic stressors are well documented (Joly-Tonetti and Lamartine, 2012), and IR-related regulatory pathways have been intensively investigated due to its importance in human medicine and as a key stressor in spaceflight (Wagner-Ecker et al., 2010; Girardi et al., 2012; Joly-Tonetti et al., 2013). By contrast, the role of miRNAs in plants under IR exposure remains largely unexplored (Cimini et al., 2019; Roy Chowdhury and Basak, 2019; Gualtieri et al., 2021; Macovei et al., 2021).

Given the overlap between the response pathways activated by IR and other abiotic stressors (Duarte et al., 2023; Mishra et al., 2024), IR-responsive miRNAs may contribute to the regulation of antioxidant pathways, DNA repair mechanisms and cell cycle control, thereby shaping the overall stress process. **Table 1** summarizes miRNAs differentially expressed in rice plants upon exposure to ionizing radiation, as well as those associated with oxidative stress and DNA damage responses.

**Table 1:** Differentially expressed miRNAs in rice in response to ionizing radiation (IR), stress-induced DNA damage response (DDR) and oxidative stress (ROS).

miRNA family	IR	DDR	ROS	Validated target(s)	References
miR156	Up/down^*^	Up		*SPL2, SPL3, SPL7, SPL9, SPL11, SPL12, SPL13, SPL14, SPL16, SPL17, HK5*	(Zhang et al., 2011; Zhang et al., 2021; Li et al., 2018)
miR159		Up		*GAMYB, GAMYBL1*	(Zhang et al., 2021)
miR160	Down	Up		*ARF8, ARF10, ARF13, ARF18, ARF22*	(Li et al., 2018; Zhang et al., 2021)
miR162		Up		*DCL1, TRE1*	(Zhang et al., 2021)
miR164	Up/down^⁑^	Up		*OMTN1 - OMTN6, DBH, PSK3*	(Zhang et al., 2011; Zhang et al., 2021; Macovei and Tuteja, 2012; Macovei and Tuteja, 2013)
miR166	Down	Down		*HOX9, HOX10, HOX32, HOX3, SGT1, EIN2.1*	(Li et al., 2018; Zhang et al., 2021)
miR167		Down		*ARF8, ARF12, ARF17, ARF25, MSH2*	(Zhang et al., 2021)
miR169		Down	Up	*NF-YA1, NF-YA2, NF-YA3, NF-YA4, NF-YA6, NF-YA10, NF-YA11*	(Li et al., 2011; Zhang et al., 2021)
miR171		Down		*HAM1 - HAM4, BGLU11, MSH3*	(Zhang et al., 2021)
miR319			Down	*PCF5, PCF6, PCF8, TCP21, GAMYB*	(Li et al., 2011)
miR395		Down		*APS1, SULTR2;1, SULTR2;2*	(Zhang et al., 2021)
miR396		Up		*GRF3, GRF4, GRF6, GRF8, GRF10, TAF6*	(Zhang et al., 2021)
miR397		Up	Up	*LAC15*	(Li et al., 2011; Zhang et al., 2021)
miR398	Up	Up		*CSD1, CSD2, CCS, SODCC2, SBP*	(Li et al., 2018; Zhang et al., 2021)
miR399	Down			*LTN1*	(Li et al., 2018)
miR408	Up	Down	Up	*UCL7, UCL8, UCL9, UCL30, UCL31, IAA6, E2F3, ERF7, ERF106, LPR1, RDD4, DSHCT*	(Li et al., 2011; Macovei and Tuteja, 2012; Macovei and Tuteja, 2013; Zhang et al., 2021)
miR414	Up			*ABP, ALT1*	(Macovei and Tuteja, 2013)
miR528			Down	*UCL23, D3, AAO2, SOD4, RFI2, LPR5, ZIP14*	(Li et al., 2011; Liu et al., 2015b; Wu et al., 2017b)
miR529			Up	*SPL2, SPL4, SPL7, SPL14, SPL16, SPL17, SPL18*	(Yue et al., 2017)
miR535		Up		*SPL4, SPL7, SPL11, SPL12, SPL14, SPL16, SPL19*	(Zhang et al., 2021)
miR810		Up			(Zhang et al., 2021)
miR827			Up	*SPX-MFS1, SPX-MFS2*	(Li et al., 2011)
miR1320	Up			*ERF096, SHL2*	(Li et al., 2018)
miR1425			Up	*PPR6, PPR9, RF1B*	(Li et al., 2011)
miR1846	Down				(Li et al., 2018)
miR2106		Up		*qSHS5*	(Zhang et al., 2021)
miR6250	Up				(Li et al., 2018)

Rows represent individual miRNA families, and columns represent stress conditions (i.e., IR, DDR and ROS). Colors indicate miRNA expression responses: yellow, upregulated; purple, downregulated; green, condition-dependent regulation; gray, no reported response. Experimentally validated target genes for each miRNA family are listed. **Supplementary Table 1** extends this analysis, providing insights into shared miRNA-mediated regulatory mechanisms across other stress types (i.e., drought, salt, cold, heat, heavy metals, nutrient deficiencies). Relevant literature sources are provided in the References section.

* Different regulation associated with irradiation.

⁑ Different regulation associated with irradiation in combination with plant developmental stage.

### 5.1 Ionizing radiation

A limited number of studies have investigated the involvement of miRNAs in the response to IR stress in species such as *A. thaliana* (Kim et al., 2016), wheat (Tondepu et al., 2024), *Tradescantia* (Subburaj et al., 2017), and *M. sativa* (Javed et al., 2019). In rice, microarray analysis of seedlings acutely exposed to 20 Gy of ^12^C heavy-ion radiation (10 Gy/min) identified significant upregulation of miR156 and miR164 families, accompanied by downregulation of their target genes, belonging to two transcription factor families, SQUAMOSA PROTEIN BINDING-LIKE (*SPL*) and NAM/ATAF/CUC (*NAC*), respectively (**Table 1**; **Supplementary Table 1**) (Zhang et al., 2011). These transcription factors are known to control plant growth and development, and their downregulation correlated with the marked decrease in seedlings height, survival rate and delayed flowering observed after heavy-ion radiation stress (Zhang et al., 2011).

The expression of miR164e, miR408 and miR414, was further analyzed in seedlings grown from seeds irradiated with different dose rates of γ-rays (^60^Co source): low dose rates (LDR) (25 and 50 Gy at 0.28 Gy/min) and high dose rates (HDR) (50, 100 and 200 Gy at 5.15 Gy/min), respectively (Macovei and Tuteja, 2013). In 5-day-old seedlings, LDR promoted the upregulation of both miR164e and miR414 (50 Gy), while HDR induced miR164e (50 Gy), miR414 and miR408 (100 and 200 Gy) (Macovei and Tuteja, 2013). In 20-day-old seedlings, miR164e was still upregulated at LDR but significantly downregulated at HDR (100 and 200 Gy), miR408 was only upregulated at LDR, and miR414 was still upregulated at both LDR and HDR (except 100 Gy) but to a lesser extent (Macovei and Tuteja, 2013). The predicted targets of miR164e, miR408 and miR414 are three putative helicase genes (*OsDBH*, *OsDSHCT*, and *OsABP*, respectively), involved in a wide range of DNA repair and replication processes (Macovei and Tuteja, 2013). Overall, although a dose-dependent increase in DNA damage and ROS accumulation was observed under the tested conditions, no clear dose- and time-dependent patterns for miRNA expression could be derived, highlighting the need for further studies to better characterize the complex mechanisms activated in plants under IR exposure (Macovei and Tuteja, 2013).

Finally, high-throughput sequencing of small RNAs in rice shoots obtained from seeds acutely exposed to low-energy N^+^ ion radiation (30 keV at 1 × 10^17^, 1.5 × 10^17^ and 2 × 10^17^ N^+^ cm^-2^) showed that 28 known miRNAs were differentially expressed, of which 12 were upregulated and 16 were downregulated (Li et al., 2018). Among these, RT-qPCR analyses of the most abundant reads confirmed the upregulation of 4 miRNA families (miR156, miR398, miR1320, and miR6250) and the downregulation of 5 miRNA families (miR156, miR160, miR166, miR399 and miR1846). Interestingly, the upregulation of miR398, accompanied by decreased transcript levels of its target gene *OsSODCC2*, which encodes a cytosolic superoxide dismutase, suggests a higher susceptibility of rice seedlings to ROS damage, in contrast to the expected induction of this antioxidant enzyme in response to irradiation. Although apparently unexpected, high doses of γ-radiation have previously been reported to suppress antioxidant responses in rice (Macovei et al., 2014). Overall, these findings suggest that miRNA/target modules may play an important regulatory function in ROS scavenging of rice seedlings under low-energy N^+^ ion radiation (Li et al., 2018).

### 5.2 DNA damage

DNA damage is widely regarded as the primary direct consequence of IR exposure (**Figure 3**), as unrepaired or misrepaired lesions can lead to permanent genetic changes, chromosomal abnormalities, altered genotype and phenotype, or even cell death (Zhang et al., 2021). Accordingly, various DNA repair mechanisms have evolved to protect the organisms against DNA damage, such as base excision repair (BER), nucleotide excision repair (NER), mismatch repair (MMR), non-homologous end joining (NHEJ), and homologous recombination (HR) (Duarte et al., 2023). In addition, miRNA-dependent post-transcription and post-translational target regulation have recently been implicated, directly or indirectly, in the DNA damage response and DNA recombination and repair in several plant species including rice (Macovei and Tuteja, 2012; Macovei and Tuteja, 2013; Zhang et al., 2021), *A. thaliana* (Zhou et al., 2007; Yao et al., 2010; Oliver et al., 2014), wheat (Akdogan et al., 2016; Sun et al., 2018a), switchgrass (Xie et al., 2010), *M. truncatula* (Gualtieri et al., 2021), *Brachypodium distachyon* (Lv et al., 2016) and *Citrus sinensis* (Liang et al., 2017).

In rice, high-throughput transcriptome sequencing analysis of seedlings treated with 10 μg/mL bleomycin identified 150 differentially expressed miRNAs in response to this DNA damaging agent, relative to untreated plants (**Table 1**; **Supplementary Table 1**) (Zhang et al., 2021). Among these, ten conserved miRNA families (miR156, miR159, miR160, miR162, miR164, miR396, miR397, miR398, miR535, and miR810) were upregulated and six miRNA families (miR166, miR167, miR169, miR171, miR395 and miR408) were downregulated after bleomycin treatment. RT-qPCR analyses confirmed contrasting patterns between several miRNAs and their target genes, in particular between miR2106 and *LOC_Os05g10980* [encoding a REX1 DNA repair family protein (Lu et al., 2025)], miR171i and *OsMSH3* (encoding a MSH DNA mismatch repair protein), and miR167h and *OsMSH2* (encoding a MSH DNA mismatch repair protein) (Zhang et al., 2021) (**Table 1**; **Supplementary Table 1**).

Consistent with the overlap between abiotic-stress and genotoxic-response pathways, additional evidence suggests that non-IR stressors may also affect repair-associated genes through miRNA-mediated regulation. For example, early salinity stress altered the expression of miR408, miR414 and miR164e, together with inverse expression of their respective target genes, *OsDSHCT*, *OsABP* and *OsDBH*, three DEAD-box helicases implicated in DNA repair- and replication-related processes (Macovei and Tuteja, 2012).

### 5.3 Oxidative stress

Reactive oxygen species, such as hydrogen peroxide, superoxide radicals, and hydroxyl radicals, are natural by-products of cellular metabolism, arising from electron transport chains and aerobic metabolic pathways in chloroplasts, mitochondria, and peroxisomes (Das and Roychoudhury, 2014; Xia et al., 2015; Xie et al., 2019). However, ionization of water molecules during IR exposure also generates ROS, and represents a major source of indirect radiation damage (**Figure 3**) (Le Caër, 2011). Although often associated with cellular damage, ROS are crucial signaling molecules involved in hormone crosstalk and in the regulation of key physiological processes, including stress responses and plant development from germination to senescence (Das and Roychoudhury, 2014; Xia et al., 2015; Xie et al., 2019). Thus, cellular ROS levels depend on the dynamic interplay between ROS generation and detoxification (Duarte et al., 2023). Under stress conditions, particularly IR stress, this equilibrium is severely disrupted, leading to excessive ROS accumulation and the onset of oxidative stress (Duarte et al., 2023). Oxidative stress can in turn cause further DNA base modification and single-strand breaks, amino acid modification and protein primary structure fragmentation, and lipid peroxidation (Sharma et al., 2012; Das and Roychoudhury, 2014; Poetsch, 2020). To mitigate these effects, organisms rely on the antioxidant defense system, consisting of enzymatic components (e.g. ascorbate peroxidase, catalase, glutathione peroxidase and superoxide dismutase) and non-enzymatic molecules (e.g. ascorbate, glutathione, tocopherol, carotenoids and proline) (Sharma et al., 2012; Xia et al., 2015; Huang et al., 2019). Genes encoding many of these antioxidant components have been reported to be under miRNA control in various plant species, such as rice (Li et al., 2011; Liu et al., 2015b; Wu et al., 2017b; Yue et al., 2017), *A. thaliana* (Jagadeeswaran et al., 2014; Wang et al., 2014a; Kayıhan et al., 2016; Li et al., 2017b), *B. napus* (Zhang et al., 2013), *V. vinifera* (Leng et al., 2017), wheat (Feng et al., 2014; Akdogan et al., 2016; Li et al., 2017a), maize (Zhang et al., 2008), *B. distachyon* (Lv et al., 2016) and *M. truncatula* (Trindade et al., 2010).

In rice, high-throughput transcriptome sequencing of H_2_O_2_-treated seedlings identified seven H_2_O_2_-responsive miRNA families, of which five were upregulated (miR169, miR397, miR408, miR827, and miR1425) and two were downregulated (miR319 and miR528) (**Table 1**; **Supplementary Table 1**) (Li et al., 2011). Taken together, the miRNAs induced by H_2_O_2_ may contribute to the suppression of plant growth and metabolism to provide an adequate response to oxidative stress. For example, upregulation of miR169, followed by downregulation of the *OsNF-YA* transcription factor family, may slow developmental and metabolic processes such as cell differentiation and respiration (Li et al., 2011). miR397-mediated downregulation of laccases might limit non-essential biological processes to conserve energy under adverse stress conditions (Li et al., 2011). Induction of miR827 and miR408 might reduce the transport of nutrients such as monosaccharides and phosphate to limit nutrient consumption (Li et al., 2011).

On the other hand, H_2_O_2_-mediated downregulation of miR319 might result in increased programmed cell death, consistent with previous results showing that H_2_O_2_ induces apoptosis (Gechev and Hille, 2005; Li et al., 2011). Furthermore, downregulation of the monocot-specific miR528 may help regulate indole-3-acetic acid (IAA) homeostasis by controlling IAA release under H_2_O_2_ stress (Li et al., 2011). Overexpression of miR528 reduced the expression of *OsAAO2*, which is responsible for the apoplastic oxidation of AsA, thereby reducing its effectiveness in detoxifying ROS (Wu et al., 2017b). Notably, plants overexpressing miR528 displayed higher redox status, suggesting that redox activity and AsA levels are inversely correlated with *OsAAO2* levels. In addition, basal ROS levels (i.e. accumulation of superoxide and hydrogen peroxide) were lower in miR528-overexpressing lines than in WT plants (Wu et al., 2017b). Plants overexpressing miR528 were also found to be more sensitive to As(III) than WT plants, likely due to a strong alteration in antioxidant enzyme activity and amino acid profiles, as well as impairment of As(III) uptake, translocation and tolerance systems (Liu et al., 2015b). Overall, these results support a key role of miR528/target genes module in the regulation of basal ROS levels in rice.

Finally, miR529a, was also induced under H_2_O_2_ stress (Yue et al., 2017). Overexpression of miR529a enhanced plant tolerance to oxidative stress, resulting in increased seed germination rate and root tip cell viability and reduced leaf rolling rate (Yue et al., 2017). Silencing its targets, *OsSPL2* and *OsSPL14*, in miR529a overexpressing plants may underlie this improved oxidative stress tolerance by regulating the expression of two downstream oxidative stress-responsive genes, *OsSOD* and *OsPOD* (Yue et al., 2017).

## 6 Conclusions and future perspectives

As unfavorable environmental and climatic conditions continue to negatively affect rice growth and yield, it is increasingly clear that miRNAs contribute to abiotic stress tolerance by safeguarding genome integrity, coordinating development and metabolism, and fine-tuning plant stress responses. In this review, we summarized current knowledge regarding rice miRNAs implicated in drought, high salinity, temperature extremes, heavy metals contamination and nutrient deficiencies, highlighting a recurring regulatory logic: stress-responsive miRNAs often repress negative regulators of stress responses, whereas stress-suppressed miRNAs can relieve repression of positive regulators of stress tolerance.

Beyond individual stress categories, the literature points to a limited set of recurrent miRNA modules that are redeployed across multiple abiotic contexts in rice. In particular, families such as miR169, miR396, miR397, miR408 and miR528 repeatedly emerge in response to distinct stresses, suggesting that rice relies on partially shared regulatory hubs along with independent stress-specific pathways. These recurrent modules converge on key processes including ROS homeostasis, nutrient allocation, hormone-related signaling, and the balance between growth and stress acclimation. At the same time, the direction and magnitude of regulation often remain stress-, tissue-, developmental stage-, and genotype-dependent, indicating that the same miRNA family can be involved in different regulatory contexts.

Despite the growing catalogue of stress-responsive miRNAs in rice (**Figure 1**; **Supplementary Table 1**), their functional roles in abiotic stress responses remain insufficiently resolved. A first bottleneck is incomplete target prediction and validation for a substantial fraction of miRNAs, which limits mechanistic interpretation. While *in silico* prediction has improved (e.g., TargetFinder (Bo and Wang, 2005), psRNATarget (Dai et al., 2018), and TAPIR (Bonnet et al., 2010)), as well as the recent development of a multi-tool approach that integrates predictions from four different algorithms (Suddal et al., 2024), experimental validation remains essential to establish *bona fide* miRNA/target interactions, for example via degradome sequencing, RNA immunoprecipitation followed by sequencing (RIP-seq), or RNA Ligase-Mediated Rapid Amplification of cDNA Ends (5’-RLM RACE). A second bottleneck is the limited, spatial and temporal resolution of available datasets. Many studies to date focus on single stress conditions, often at a single time point and predominantly during the early seedling stage. This leaves major sources of biological variation unexplored, including tissue-, developmental stage, and cultivar-specificity, as well as stress intensity, duration, recovery, and multiple stress combinations. This is particularly important because isolated single-stress assays poorly reflects the complexity of natural and agricultural environments and may capture only part of the relevant miRNA/target regulatory networks. Transgenerational persistence of stress responses involving miRNAs remains to be tested (Holeski et al., 2012), yet is likely relevant for stress memory and long-term adaptations.

Finally, ionizing radiation remains underrepresented as an abiotic stress in plant miRNA research. However, as the field shifts from a largely human-centric perspective toward the environmental relevance of anthropogenic activities, there is a growing need to understand how genotoxic effects impact ecosystems. In rice, evidence is still largely restricted to acute laboratory exposures, but the overlap of IR-responsive miRNAs with DNA repair (4 miRNAs) and oxidative stress pathway (1 miRNA) points to broader, interconnected multi-stress regulatory networks (**Table 1**). Given that ROS-mediated signaling is shared across many abiotic stresses, integrating multi-stressors designs will be a key to separating shared from stress-specific miRNA modules and to clarifying whether ROS act as a central regulatory hub shaping miRNA-driven gene regulatory networks.
